# Predictive factors for a one-year improvement in nontuberculous mycobacterial pulmonary disease: An 11-year retrospective and multicenter study

**DOI:** 10.1371/journal.pntd.0005841

**Published:** 2017-08-07

**Authors:** Gilbert Cadelis, Rodolphe Ducrot, Arnaud Bourdin, Nalin Rastogi

**Affiliations:** 1 Department of Pulmonary Medicine, University Hospital of Pointe-à-Pitre, Pointe-à-Pitre, Guadeloupe, France; 2 Department of Pulmonary Medicine, University Hospital of La Reunion, Saint Pierre, La Reunion, France; 3 Department of Respiratory Diseases, University Hospital of Montpellier, Montpellier, France; 4 PhyMedExp, University of Montpellier, INSERM U1046, CNRS UMR 9214, Montpellier, France; 5 WHO Supranational TB Reference Laboratory, Tuberculosis & Mycobacteria Unit, Institut Pasteur de la Guadeloupe, Abymes, Guadeloupe, France; University of Tennessee, UNITED STATES

## Abstract

**Background:**

Nontuberculous mycobacterial pulmonary disease (NTM-PD) has become an emerging infectious disease and is responsible for more deaths than tuberculosis in industrialized countries. NTM-PD mortality remains high in some series reportedly ranging from 25% to 40% at five years and often due to unfavorable evolution of NTM-PD despite established treatment. The purpose of our study was to search for early factors that could predict the favorable or unfavorable evolution of NTM-PD at the first year of treatment.

**Methods:**

In this retrospective and multicenter study, we selected 119 patients based on clinical, radiological and microbiological data from 2002 to 2012 from three French university hospitals (Guadeloupe, Martinique, Montpellier) with definite (meeting the criteria of the American Thoracic Society and the Infectious Disease Society of America in 2007; ATS/IDSA) or probable (one positive sputum culture) NTM-PD. We compared two patient groups: those who improved at one year (clinical symptoms, radiological lesions and microbiology data) and those who did not improve at one year. The data were analyzed for all patients as well as for subgroups by gender, HIV-positive patients, and *Mycobacterium avium complex* (MAC) infection.

**Results:**

The average patient age was 50 years ± 19.4; 58% had respiratory comorbidities, 24% were HIV positive and 19% had cystic fibrosis. Coughing concerned 66% of patients and bronchiectasis concerned 45%. The most frequently isolated NTM were MAC (46%). 57% (n = 68) of patients met the ATS criteria and improved status concerned 38.6% (n = 46). The improvement factors at one year of NTM-PD were associated with the duration of ethambutol treatment: (Odds ratio adjusted [ORa]: 2.24, 95% Confidence interval [CI]; 2.11–3.41), HIV-positive status: (ORa: 3.23, 95% CI; 1.27–8.45), and male gender: (ORa: 2.34, 95% CI; 1.26–8.16). For the group with NTM-PD due to MAC, improvement was associated with the duration of macrolide treatment (ORa: 3.27, 95% CI; 1.88–7.30) and an age <50 years (ORa: 1.88, 95% CI; 1.55–8.50).

**Conclusion:**

In this retrospective multicenter study, improvement at one year in patients with definite or probable NTM-PD was associated with the duration of ethambutol treatment, HIV-positive status and male gender. For the group of patients infected with MAC, improvement was associated with the duration of macrolide treatment and an age <50 years. Identifying predictors of improvement at one year of NTM-PD is expected to optimize the management of the disease in its early stages.

## Author summary

Early predictive factors for a favorable development of nontuberculous mycobacterial pulmonary disease (NTM-PD) are important to improve management due to the high mortality of this infection at 5 years. The purpose of this study was to search for early factors that could predict at the first year, the favorable or unfavorable evolution of NTM-PD. This multicenter and retrospective study shows the importance of the duration of use of certain antibiotics (e.g. ethambutol and macrolides) in combination with other drugs in the one-year improvement of patients with NTM-PD. It also confirms the favorable prognosis at one year of NTM-PD patients with HIV-positive status. Identifying predictors of improvement at one year of NTM-PD is expected to optimize prognosis of the disease in its early stages.

## Introduction

Infection with nontuberculous mycobacteria (NTM) preferentially affects the lungs and occurs by inhalation of aerosols containing mycobacteria [1, 2]. NTM are ubiquitous environmental bacteria found in soil, but also in other sources such as contaminated water taps. The frequency of NTM species can vary from region to region in the world [1, 3]. NTM pulmonary disease (NTM-PD) has today become an emerging infectious disease in industrialized countries. Its increasing prevalence is estimated at more than 50 cases per 100,000 persons in some demographic groups in the US [4]; while its incidence in Europe ranges from 0.2 to 2.9 / 100,000 inhabitants [1]. Remarkably, all NTM species are not likely to cause NTM-PD; only a few species such as *Mycobacterium avium complex* (MAC), *M*. *abscessus*, *M*. *xenopi* and *M*. *kansasii* are frequently involved [5]. Indeed, the clinical relevance of NTM differs by species since they are not endowed with the same virulence [6].

The diagnostic criteria of the American Thoracic Society and the Infectious Disease Society of America in 2007 (ATS/IDSA) [7] have established the diagnosis of NTM-PD based on clinical symptoms, radiological lesions and microbiology data. During this decade, real progress has been made in the understanding of this disease [4]. We know for example that besides immunosuppression by HIV or cystic fibrosis, NTM-PD occurs in lungs whose architecture is already weakened by chronic respiratory diseases such as primarily chronic obstructive pulmonary disease (COPD) and bronchiectasis [1, 5]. The establishment of NTM-PD in impaired lungs can cause the destruction of the pulmonary parenchyma [8] and eventually lead to death due to the evolution of NTM-PD [9]. Patients with NTM-PD are not all treated because current treatments are often long, expensive and not without side effects [10]. NTM-PD mortality remains high in some series ranging from 25% to 40% at five years [1, 9, 11]. The main factors of poor outcomes identified in mortality studies at five years corresponded to an advanced age, the existence of respiratory comorbidities, radiological cavity lesions, and some mycobacteria such as *M*. *xenopi* [11,12,13].

Given the deteriorating respiratory status of patients due to the evolution of NTM-PD despite established treatment and the relatively high mortality at five years, it seemed important to search for early factors that could predict from the first year the favorable or unfavorable evolution of NTM-PD, and thus improve prognosis. Hence, the main purpose of this study was to identify factors that contribute to the clinical, radiological and microbiological improvement at one year of a cohort of 119 patients with definite (meeting the criteria ATS/IDSA) or probable (one positive sputum culture) NTM-PD, regardless of their immune status or their respiratory history. The secondary goal of this study was to report for the first time, a clinical, radiological and microbiological description of NTM-PD in a population of Afro-Caribbean patients in the French West-Indies.

## Materials and methods

### Ethics statement

This observational study received approval from the Institutional Review Board of the French learned society for respiratory medicine (Société de Pneumologie de Langue Française; No: 2015–003). All the participants gave their written consent. The parents/guardians provided written informed consent on behalf of participants below 18 years of age.

This study was carried out in accordance with the principles of the Helsinki Declaration.

### Study design and patient selection

This study was a retrospective, multicentric, observational study over a 11-year period between 2002 and 2012 in three French university hospitals (CHU), two of which are located in the French West-Indies (University Hospital of Fort de France, Martinique; and University Hospital of Pointe-à-Pitre, Guadeloupe), and the 3rd in Metropolitan France (University Hospital of Montpellier, France). From the computerized databases of the bacteriological laboratories of these three institutions, we searched all patients over 13 years old with at least one positive culture for NTM between 2002 and 2012. A total of 119 patients were therefore finally retained for this study regardless of their immune status. The exclusion criteria were an age below 13 years and the absence of patient consent.

### Data collected

#### Patient data

Age, sex, place of residence, tobacco and alcohol consumption, history of respiratory disease, diabetes, the presence of known gastroesophageal reflux disease, usual medications, HIV-positive serology, and taking immunosuppressants were recorded.

#### Clinical and radiographic data

NTM discovery mode, respiratory symptoms and general symptoms at diagnosis. The main thoracic radiographic abnormalities: cavities in the pulmonary parenchyma, presence or absence of solitary pulmonary nodules (>1 cm), bronchiectasis with or without micronodules, alveolar condensations, mediastinal lymph nodes (>1 cm) and pleural effusion. All patients had a thorax X-ray and chest CT scans interpreted by two radiologists specialized in thoracic imaging.

#### Bacteriological data

The sources of bacteriological samples (sputum, bronchoalveolar lavage, pleural fluids, blood cultures and surgical samples), the *Mycobacterium* species and the number of positive bacteriological culture samples were recorded. The bacteriological laboratories of the three institutions involved in the study have specialized units referenced nationally for NTM. For each patient in the study, we determined those who met the ATS/IDSA diagnostic criteria [7] for NTM-PD on the evaluation of clinical, radiological and microbiological criteria and the exclusion of other diagnoses (tuberculosis, cancer, histoplasmosis, etc.). ATS/IDSA 2007 microbiological criteria [7] are defined by the presence of at least two positive bacteriological culture samples from a non-sterile site, or at least one positive bacteriological culture sample from a sterile site.

In our series, NTM-PD was distinguished by two patient categories: those who completely met the diagnostic criteria of pulmonary disease defined by the ATS/IDSA and therefore had definite NTM-PD and those who did not meet the criteria because they had one positive sputum culture for NTM, which corresponded to probable NTM-PD cases, only meeting the ATS/IDSA clinical and radiological criteria for NTM-PD.

#### Therapeutic data

We recorded if the patient was treated or not, the type of antibiotic molecules used, combinations of therapeutic molecules, the total duration of antibiotic treatment and the treatment side effects. The therapeutic management and monitoring of patients were decided by clinicians of the three institutions in consultation with the National Mycobacteria Reference Center. The clinician in charge implemented the treatments and the follow-up of the patient that were commonly initiated in the hospital, either in hospital itself or the outpatient department depending on the clinical condition of the patient. All treated patients received a therapeutic combination.The therapeutic combinations initially proposed to the patients per NTM species isolated are summarized in S1 Table.

#### Patient follow-up

Patient follow-up was noted from the medical records. This follow-up consisted of scheduling a consultation every 2–3 months until culture negativity, and was followed three times a year throughout the duration of treatment. In the follow-up visits, there was a check of treatment adherence and eventual side effects, as well as obtaining bacteriological samples and thoracic imaging, if deemed necessary. Nonetheless, in all cases the patients benefited from a thoracic CT scan at varying frequencies during their follow-up.

#### Patient outcome at one year

The disappearance or persistence of clinical symptoms at diagnosis, changes in thoracic imaging (regression or disappearance of the initial lesions), negative or positive bacteriological samples, death and discovery of another disease during follow-up were recorded.

### Primary evaluation criteria

This was a composite endpoint defined by the disappearance at one year of respiratory symptoms and/or initial symptoms, regression or normalization at one year of the initial radiological lesions, and negative bacteriological cultures at one year. Negative bacteriological cultures were defined as at least three consecutive negative respiratory culture specimens at the end of one year. Patients were classified as having an improved status at one year only if all the three criteria were met (vs. unimproved status if this was not the case).

### Statistical analysis

Statistical analyses were designed to determine the parameters related to the primary endpoint, i.e., an improved status at one year. Univariate analysis was first conducted to study the independent variables related to the primary endpoint. Statistical tests used for categorical variables were the Chi-squared test or the Fisher exact test and for quantitative variables, the Student’s t-test or the Wilcoxon-Mann-Whitney test. For all statistical tests, the significance level was set at 5% and a power >90%.

Independent variables with a p-value less than 0.2 determined by univariate analysis were retained for the multivariate model. Multivariate analysis consisted of logistic regression analysis. The dependent variable was the binary variable (improved / unimproved status); independent variables were introduced into the model using a backward regression approach. Variables with a p-value less than 0.05 were selected. The results were produced as odds ratios with 95% confidence intervals.

The choice of multivariate logistic regression was dictated:

- By the characteristics of the variables to be studied. The dependent variable was binary and was used to separate two groups of individuals: those who improved according to the main criterion and those who did not improve. The explanatory variables were both quantitative and qualitative.
- By the study objective, which was to search for factors and their weight among the adjusted variables (adjusted odds ratio) that could influence the variable to be explained while adjusting for the confounding factors.

A subgroup analysis was performed for the population infected by MAC for the HIV-positive population and by gender.

Processing and statistical analysis were performed using version 3.3.2 of the R software. The libraries used in the statistical analysis with R included: base-package, stats-package, BioStatR-package, MASS-package and pwr-package.

## Results

### General characteristics of NTM-PD patients with improved and unimproved status (Table 1)

**Table 1:** The general characteristics of patients with improved versus unimproved status at one year.

	Total patients N = 119 (%)	Patients with improved status n = 46 (38.6%)	Patients with unimproved status n = 73 (61.3%)	P value
***Age (mean ± SD)*,*years***	50.4 ±-19.4	46.1 ±19.0	55.0 ±20.1	0.05
***Sex***				0.06
*Male*	80(67.2)	36 (78.2)	44 (60.2)	
*Female*	39(32.7)	10 (21.7)	29 (39.7)	
***Place of residence***				0.01
*Guadeloupe (West Indies)*	56(47.0)	27 (58.6)	29 (39.7)	
*Martinique (West Indies)*	19(15.9)	2 (4.3)	17(23.2)	
*Montpellier (France)*	44(36.9)	17 (36.9)	27 (36.9)	
***Respiratory history***				0.17
*yes*	70(58.8)	23 (50.0)	47 (64.3)	
*No*	49(41.1)	23 (50.0)	26 (35.6)	
***Respiratory disease***				0.27
*-Cystic fibrosis*	23(19.3)	11 (23.9)	12 (16.4)	
*-Bronchiectasis*	23(19.3)	7 (15.2)	16 (21.9)	
*-Chronic obstructive pulmonary disease*	18(15.1)	2 (4.3)	16 (21.9)	
*-Lung fibrosis*	2(1.6)	0 (0.0)	2 (2.7)	
*-Asthma*	2(1.6)	2 (4.3)	0 (0.0)	
*-previous history of tuberculosis*	3(2.5)	2(4.3)	1 (1.3)	
*-kyphoscoliosis*	1 (0.8)	1 (2.1)	0 (0.0)	
*-Sarcoidosis*	1 (0.8)	0 (0.0)	1 (1.3)	
***Immunosuppressive therapy***	10(8.4)	2 (4.3)	8 (10.9)	0.36
***HIV serology positive***	29(24.4)	17(36.9)	12(16.4)	0.02
*CD4<200/mm3*	23(19.3)	14(30.4)	9(12.3)	0.02
***Diabetes***	17(14.3)	7(15.2)	10(13.6)	0.66
**Gastroesophageal reflux**	15(12.6)	7 (15.2)	8 (10.9)	0.30
***Smoking***	38(31.9)	12 (26.0)	26(35.6)	0.46
***Alcoholism***	22(18.5)	12(26.0)	10(13.6)	0.14
**ATS/IDSA criteria**	68(57.1)	27(58.6)	41(56.1)	0.93

**Improved status at 1-year**: Patients improved clinically and radiological and their microbiological samples were negative. **Unimproved status at 1-year**: The patients did not improve their clinical state or their radiological lesions or their microbiological samples did not negative.

**ATS/IDSA criteria**: American Thoracic Society and the Infectious Disease Society of America

Patients with an improved status represented 38.6% (n = 46). The regression of clinical symptoms at one year concerned 56.3% (67/119), the disappearance or regression of radiological lesions at one year concerned 38.6% (46/119) of patients and negative bacteriological cultures at one year were obtained for 51.2% (61/119). A statistically significant difference was revealed between the two groups for age (p<0.05), place of residence (p<0.01) and the percentage of patients with HIV-positive serology (p<0.02).

No difference was found between the two groups for the ATS/IDSA diagnostic criteria (58.6% vs. 56.1%, p = 0.93).

### Clinical and radiological characteristics of NTM-PD patients with improved and unimproved status (Table 2)

**Table 2:** Clinical and radiological characteristics of patients enrolled.

	Total patients N = 119 (%)	Patients with improved status N = 46 (38.6%)	Patients with unimproved status n = 73 (60.3%)	P value
**Circumstances of discovery**				0.006
Respiratory symptoms	40 (33.6)	18 (39.1)	22 (30.1)	
Alteration of general state	20 (16.8)	11 (23.9)	9 (12.3)	
Hemoptysis	15 (12.6)	10 (21.7)	5 (6.8)	
Systematic review	14 (11.7)	10 (21.7)	4 (5.4)	
Hyperthermia	14 (11.7)	7 (15.2)	7 (9.5)	
Radiologic abnormalities	11 (9.2)	8(17.3)	3 (4.1)	
Extra-pulmonary signs	5 (4.2)			
Skin lesions		2 (4.3)	1 (1.3)	
Cervical abscess		1(2.1)	0(0.0)	
Paraspinal abscess		1(2.1)	0(0.0)	
**Symptoms at diagnosis**				
Cough	79 (66.4)	32 (69.5)	47 (64.3)	0.59
Sputum	61 (51.3)	24 (52.1)	37 (50.6)	0.60
Weight loss	60 (50.4)	26 (56.5)	34 (46.5)	0.52
Asthenia	55 (46.3)	26 (56.5)	29 (39.7)	0.32
Hyperthermia	43 (36.1)	15 (32.6)	28(38.3)	0.66
Dyspnea	48 (40.3)	15 (32.6)	33 (45.2)	0.47
Hemoptysis	22 (18.5)	9 (19.5)	13 (17.8)	0.67
**Radiological pattern**				
Bronchiectasis	54 (45.4)	19 (41.3)	35 (47.9)	0.73
Nodular opacities (>1cm)	24 (20.1)	6 (13.0)	18 (24.6)	0.24
Cavities	26 (21.8)	8 (17.3)	18 (24.6)	0.50
Consolidation	45 (37.8)	18 (39.1)	27 (36.9)	0.54
Mediastinal lymphadenopathy (>1cm)	31 (26.1)	10 (21.7)	21 (28.7)	0.54
Pleural effusion	14 (11.8)	6 (13.0)	8 (10.9)	0.77

**Improved status at 1-year**: Patients improved clinically and radiological and their microbiological samples were negative. **Unimproved status at 1-year**: The patients did not improve their clinical state or their radiological lesions or their microbiological samples did not negative.

A statistically significant difference was found between the two groups (improved / unimproved status) in the circumstances of the disease discovery (p<0.006). There was no statistically significant difference between the two groups in terms of initial respiratory symptoms and initial radiological lesions.

### Bacteriological characteristics of NTM-PD patients with improved and unimproved status (Table 3)

**Table 3:** Bacteriological characteristics of the patients.

	Total Patients n = 119	Patients with improved status n = 46 (38.6%)	Percentage of patients treated and improved (%)	Patients with unimproved status n = 73(61.3%)	P Value
***Mycobacterium species***					0.25
*M*. *avium complex*	55 (46.2)	26 (56.5)	53 (18/34)	29 (39.7)	
*M*. *abscessus complex*	17 (14.3)	5(10.8)	45 (5/11)	12 (16.4)	
*M*. *fortuitum*	16 (13.4)	4 (8.6)	33 (1/3)	12 (16.4)	
*M*. *simiae*	11 (9.2)	7 (15.2)	80 (4/5)	4 (5.4)	
*M*. *gordonae*	8 (6.7)	3 (6.5)	66 (2/3)	5 (6.8)	
*M*. *kansasii*	5 (4.2)	1 (2.1)	20 (1/4)	4(5.4)	
*M*. *xenopi*	3 (2.5)	3 (6.5)	100 (3/3)	0 (0.0)	
*M*. *chelonae*	1 (0.8)	1 (2.1)	100 (1/1))	0 (0.0)	
*M*. *gilvum*	1 (0.8)	1 (2.1)	100 (1/1)	0 (0.0)	
*M*. *szulgai*	1 (0.8)	0 (0.0)	0 (0/1)	1 (1.3)	
*M*. *genavense*	1 (0.8)	1 (2.1)	100 (1/1)	0 (0.0)	
***Source of positive culture***:					
***-1 positive sputum culture***	43 (36.1)	16 (34.7)		27 (36.9)	0.11
***->2 positive sputum cultures***	41 (34.4)	14 (30.4)		27 (36.9)	0.70
***-Bronchoalveolar lavage culture***	19 (15.9)	10 (21.7)		9 (12.3)	0.31
***-Lung biopsy + sputum culture +***	8 (6.7)	3 (6.5)		5 (6.8)	0.10
***-Blood culture***	7 (5.8)	3 (6.5)		4 (5.4)	0.11
***-Pleural effusion culture***	1 (0.8)	0		1 (1.3)	0.39
-ATS /IDSA microbiologic criteria*	76 (63.9)	30 (65.2)		46 (63.0	0.96

**Improved status at 1-year**: Patients improved clinically and radiological and their microbiological samples were negative. **Unimproved status at 1-year**: The patients did not improve their clinical state or their radiological lesions or their microbiological samples did not negative. **The P Value** results from the comparison between these two groups (Improved/unimproved)

**ATS/IDSA**: American Thoracic Society and the Infectious Disease Society of America

**Percentage of patients treated and improved =** number of patients treated and improved for the species / all patients treated for the same species

In Guadeloupe, the main NTM encountered in decreasing order were MAC, *M*. *simiae* and *M*. *fortuitum*, in Martinique, *M*. *fortuitum* followed by MAC, then M. *gordonae*; and in Montpellier, MAC then *M*. *abscessus* complex, followed by *M*. *xenopi*. No statistically significant difference was found between the improved / unimproved status groups for the mycobacterial species. The ATS/IDSA criteria were met for 62% of patients with MAC, 82% with *M*. *abscessus*, 50% with *M*. *fortuitum* and 45% with *M*. *simiae*. For bacteriological samples, 76% met the ATS/IDSA microbiological criteria. There was no statistically significant difference between the two groups for the ATS/IDSA microbiological criteria. The positive predictive value (PPV) of the ATS/IDSA microbiological criteria for definite NTM-PD was 89% (68/76) CI 95% (83%-94%). Lastly, patients who did not meet the ATS microbiological had a four-fold increased risk of death at one year (OR = 4.01, 95% CI; 1.40–14.51, p<0.01).

### Treatment characteristics (molecules and duration of treatment) received by NTM-PD patients with improved and unimproved status and patient outcomes (Table 4)

**Table 4:** Therapeutic characteristics of the patients.

	Total patients n = 119 (%)	Patients with improved status n = 46 (38.6%)	Patients with unimproved status n = 73 (61.3%) n =	P value
Patients treated				0.19
Yes	63 (52.9)	30 (65.2)	33 (45.2)	
No	56 (47.1)	16(34.7)	40 (54.7)	
*Duration of treatment (months)*	9.1 ±6,0	10.6 ± 5.8	8.5 ± 6.3	0.10
Macrolides (Clarithromycin or Azithromycin)		6.4± 6.2	4.5 ± 4.6	0.25
Ethambutol		6.0 ±5.9	2.46 ±2.8	0.001
Rifampicin		5.6 ± 5.9	4.1 ± 3.5	0.34
Isoniazid		3.5 ±4.8	3.1 ± 4.3	0.93
Moxifloxacin		1.9 ± 4.2	1.2 ±3.5	0.83
Rifabutin		1.9 ± 3.9	1.1 ± 2.6	0.38
Pyrazinamide		0.7 ±1.1	1.7 ± 3.1	0.21
Aminoglycoside (Amikacin)		1.2 ±1.8	0.9 ±1.8	0.78
Meropenem		0.3±1.2	0.7 ± 1.6	0.25
Negative cultures at one year	61 (51.2)	46(100)	15(20.5)	0.001
Patient outcome				
One-year mortality	17 (14.2)	0.0 (0)	17 (23.2)	0.001

**Improved status at 1-year**: Patients improved clinically and radiological and their microbiological samples were negative. **Unimproved status at 1-year**: The patients did not improve their clinical state or their radiological lesions or their microbiological samples did not negative.

No statistically significant difference was found between the two groups for treated patients, as well as in the total duration of treatment. There was a statistically significant difference in the duration of ethambutol treatment between the two groups (p<0.001, effect size: 0.81, power: 0.99). Side effects related to treatment concerned 10 of 63 patients (15.8%), five had minor side effects (digestive disorders) and five had major side effects (three cases of drug-induced hepatitis, one case of eye damage and a kidney failure). These 5 patients with major side effects had to stop their therapy. No patient in our cohort benefited from associated surgical treatment.

There was a statistically significant association between the absence of negative cultures and mortality at one year (p<0.001). The conversion rate of bacterial cultures was 60% (33/55) for MAC, 35% (6/17) for *M*. *abscessus complex*, 37% (6/16) for *M*. *fortuitum* and 72% (8/11) for *M*. *simiae*.

The total number of mortalities at one year was 14.2% (n = 17), all belonging to the unimproved group. The average age of deceased patients (13 men and four women) was 60 years±12.7. We recorded 52% tobacco smokers, and 44% COPD, 29% HIV-positive and 5% cystic fibrosis patients. NTM of deceased patients were MAC (9/17; 52.9%), *M*. *abscessus complex* (4/17; 23.5%), *M*. *kansasii* (2/5; 40%) and *M*. *fortuitum* (2/16; 12.5%). Eight patients died of unfavorable NTM-PD evolution, one patient from pulmonary embolism and two patients from COPD exacerbations. An association between the mortality rate and mycobacterial species in the study (p = 0.86) was not found.

### Factors associated with NTM-PD patient improvement at one year by multivariate analysis (Table 5)

**Table 5:** Factors associated with improved status at one year, multivariate analysis.

Variables	Odds Ratio	confidence interval at 95%	*P val*u*e*
**For all patients (n = 119)**			
HIV positive	3.23	1.27–8.45	0.01
Male	2.34	1.26–8.16	0.02
Duration of the treatment by Ethambutol (months)	2.24	2.11–3.41	0.001
**For non–HIV-positive patients (n = 90)**			
Male	3.54	1.12–14.0	0.04
Duration of the treatment by Ethambutol (months)	1.90	1.50–3.50	0.001
**For patients with complete ATS/IDSA criteria (n = 68)**			
Duration of the treatment by Ethambutol (months)	2.45	1.82–7.45	0.01
**For non-HIV-positive patients with complete ATS/IDSA criteria (n = 55)**			
Duration of the treatment by Ethambutol (months)	1.85	1.55–6.53	0.001
**For patients with MAC (n = 55)**			
Age <50ans	1.88	1.55–8.50	0.02
Duration of the treatment by Macrolide (months)	3.27	1.88–7.30	0.003
**For non-HIV positive patients with MAC (n = 38)**			
Duration of the treatment by Macrolide (months)	2.88	2.15–8.56	0.007

**ATS/IDSA**: American Thoracic Society and the Infectious Disease Society of America

**MAC**: Mycobacterium *avium* complex

Factors associated with an improvement at one year were the male gender (OR = 2.34), HIV-positive serology (OR = 3.23) and duration of ethambutol treatment (OR = 2.24). For the population meeting the ATS/IDSA diagnostic criteria, the factor associated with improvement was the duration of ethambutol treatment (OR = 2.45). For the group of patients infected by MAC, improvement factors were associated with age under 50 years (OR = 1.88) and duration of macrolide treatment (OR = 3.27).

For the group of non HIV-positive patients, improvement factors were associated with Male (OR = 3.54) and duration of ethambutol treatment (OR = 1.90).

### Characteristics and outcome of NTM-PD study patients infected with MAC and *M*. *kansasii*

- Patient group with MAC (S2 Table): Patients infected with MAC represented 46% of our sample. Cavity forms mainly concerned men (8/40; 20%). Bronchiectasis concerned 93% (14/15) of women. Deaths at one year concerned men only at 22% (9/40), eight with cavity lesions. Men smoked and had a more pronounced respiratory history than women. A statistically significant difference was found between the group of improved and unimproved patients for age (p<0.04), total treatment duration (p<0.04), duration of macrolide treatment (p<0.04, effect size: 0.94, power: 0.92), negative bacteriological cultures at one year (p<0.001) and death at one year (p<0.001).
- NTM-PD patient group with *M*. *kansasii* (n = 5): This group of patients had a significant mortality rate (40%; n = 2/5). The average age was 50 years with four males and one female. From the patient history, two patients were monitored for pulmonary fibrosis and two patients had HIV-positive serology with a CD4 count <50. Radiology revealed 3/5 patients (60%) with cavities.

### General characteristics, treatment and outcome of the NTM-PD and HIV-positive patients in the study (S3 Table)

A total of 29 patients were included. The CD4 count was below 200 for 23 patients (79%). The discovery of NTM revealed an HIV-positive status for 98% of patients. The most frequently found species was MAC (58%). A statistically significant difference between the two groups (improved / unimproved status) was found for age (p<0.04), percentage of treated patients (p<0.04), negative bacteriological cultures at one year (p<0.001), and percentage of deaths at one year (p<0.04).

Sixteen of the 29 HIV–positive patients (55%) were treated. The percentage of patients improved on treatment was 81% (13/16).

### General characteristics, treatment, and outcome of NTM-PD patients in the study analyzed by gender (S4 Table)

A statistically significant difference was found between men and women for age (p<0.04), chronic respiratory diseases (cystic fibrosis, COPD and bronchiectasis; p<0.004), the incidence of sputum for women (p< 0.02), the type of radiological lesions and improved status at one year (p<004) in favor of men.

## Discussion

In this work, HIV infection, treatment duration with ethambutol in combination with other antibiotic molecules, and the male gender were independent factors associated with a favorable outcome at one year of definite or probable NTM-PD. The composite endpoint adopted in our study could have a prognostic value as it effectively allows to discriminate patients who survived at one year from those who did not. When clinical symptoms, radiological lesions and negative microbiology data were observed in function of patient outcome at one year, only the absence of negative cultures was associated with death.

The diagnostic criteria for NTM-PD proposed by the ATS/IDSA were not associated with an improved status in our study. These results were similar to those found by other authors [11,14] on the relationships between ATS/IDSA diagnostic criteria and patient prognosis. In our study, the ATS/IDSA microbiological criteria had an excellent PPV of 89% for definite NTM-PD. Winthrop et al. [15] reported a PPV of 86% for NTM-PD. Jankovics et al. [16] showed that the PPV for NTM-PD varied from 64% to 94% depending on the clinical relevance of the NTM.

The majority of NTM-PD patients in this study (52%) were from the French West-Indies. In Guadeloupe, the species that predominated among these patients was MAC (45%), while in Martinique, it was *M*. *fortuitum* (47%). These frequencies are in agreement with Streit et al. [17] based on an epidemiological study in Guadeloupe, Martinique and French Guiana. Likewise, NTM-PD isolates from Montpellier showed a predominance of MAC (57%) followed by *M*. *abscessus* (32%), which paralleled NTM epidemiology in metropolitan France [18]. Finally, NTM-PD patients with *M*. *abscessus* in our study met more often the ATS/IDSA criteria for pulmonary disease (82%) than patients with MAC (62%). It should however be underlined that clinical characteristics and outcomes for the cohort with MAC were not further categorized by genetic sequencing to discriminate with distinct MAC species as reported recently [19], hence not allowing to conclude on a possible variability as regards to ATS/IDSA criteria for pulmonary disease depending on infection with *M*. *avium*, *M*. *intracellulare*, *M*. *chimaera*, or other MAC species.

In our study, no association between improved status and NTM species could be distinguished at one year. Indeed, the relatively shorter observation period in our study may not have allowed to perceive a species dependent association; e.g., Andrejack et al. [11] did not find significant differences in mortality based on the mycobacterial species in the first year of their study, although *M*. *xenopi* was associated with increased mortality as compared to MAC at the longer term. In this respect, mortality due to *M*. *kansasii* in our study (40%) seemed high as compared to published data [20], although our cohort (n = 5) was too small to draw conclusions. Furthermore, these patients had significant comorbidities, cavity lesions (3/5) and poor prognosis [12]. For NTM-PD with MAC, two conventional radiological presentations were the cavity radiological form that preferentially affected male smokers with a respiratory history, poor prognosis at one year, and bronchiectasis in women with no deaths at one year [12, 21]. The age <50 years and duration of macrolide treatment was associated with an improvement at one year. Advanced age was reported to be a poor prognostic factor [11,12].

Macrolides are an essential treatment of NTM-PD due to MAC [1, 4, 22]. In our group with MAC, the duration of macrolide treatment was predictive of an improvement at one year. Macrolide pharmacodynamics could explain the more effective action of these molecules over time; it was shown that the maximum eradication kinetics of MAC was slow with clarithromycin compared to amikacin [23]. Hence our observation that the duration of macrolide treatment was an improvement factor at one year for NTM-PD due to MAC was not unexpected, nonetheless it remarkably corroborates the positive effect of the duration of macrolide treatment in a clinical study. Significantly, the duration of ethambutol treatment in combination with other molecules in our study emerged as an independent factor of NTM-PD improvement at one year. Although the current treatment of NTM is largely empiric [24], ethambutol, an inhibitor of arabinogalactan synthesis, is known to significantly boost *M*. *avium* drug susceptibility in vitro [25], and was shown to enhance activity both of clarithromycin and rifampin against MAC in extra- and intracellular assays at serum level concentrations [26]. Indeed, the tripartite cell-envelope architecture of *M*. *avium* [27], is partially responsible for exclusion of antimicrobial agents, leading to its observed multiple drug resistance [28, 29]. With regard to the *M*. *avium* lipid bilayer at the surface of the cell-wall skeleton which decreases the permeability for hydrophilic molecules, ethambutol acts by decomposing not only the skeleton but also the lipid layer, thereby facilitating the diffusion of antibiotics [30]. These observations corroborate our main hypothesis that more the action of ethambutol is maintained over time, more the antibiotics are able to access and concentrate intracellularly with higher concentrations in the bacterium. This assumption is further substantiated by observations on synergistic effects of ethambutol with several molecules with intracellular action: rifampin, quinolones and macrolides [31, 32]. Undeniably, ethambutol is an important component in the current multidrug regimens for treatment of patients with MAC lung disease [7, 33, 34]; the microbiological response being significantly related to the duration of its use [35].

In our study, an HIV-positive status was an independent factor in improving NTM-PD at one year. Other studies have shown a dramatic improvement in HIV-positive patients with NTM-PD due to immune restoration by anti-HIV therapies [36]. Restoration of immunity, monitoring of these patients in specialized structures and treatment of NTM-PD have played an important role in improving prognosis.

Men with NTM-PD have been reported as having a worse prognosis in terms of mortality than women [11, 12]. In our study, men showed greater improvement at one year than women. The men were younger and sputum was more common among women (79% vs. 37%), as was bronchiectasis (64% vs. 36%). There was no difference between the sexes on the negativity of bacteriological cultures. Women showed little improvement clinically and radiologically compared to men due to the persistence of clinical symptoms. However, mortality at one year remained higher in men.

The strengths of our study are based on its multicenter approach, subgroup analyses and a large cohort constituted by the analysis of clinical and radiological records of patients rather than on discharge data. However, the limitations of this study are its retrospective design and the short observation period (one year), which does not allow to know if the improvement observed will persist over time. Another limitation of this study is the inclusion of probable cases of NTM-PD, which nonetheless translates a reality faced by clinicians. However, the current diagnostic criteria for NTM-PD does not have perfect sensitivity and specificity [37], and a fraction of these probable cases may evolve during monitoring to definite cases, although there is no data to confirm this assumption. Furthermore, at times one cannot conclude definitely, for example for the HIV subgroup, since the numbers in each sample (improved / unimproved) were too small to be able to carry out a multivariate analysis.

### Conclusions

In this retrospective multicenter study, improvement at one year in patients with definite or probable NTM-PD was associated with the duration of ethambutol treatment, HIV-positive status and male gender. For the group of patients infected with MAC, improvement was associated with the duration of macrolide treatment and an age <50 years. Identifying predictors of improvement at one year of NTM-PD is expected to optimize the management of the disease in its early stages.

## Supporting information

S1 Table Treatment of nontuberculous mycobacterial pulmonary disease (NTM-PD). (DOCX)

S2 Table The general characteristics and treatment outcome of patients infected with *Mycobacterium avium* complex (MAC). (DOCX)

S3 Table The general characteristics and treatment outcome of patients with positive HIV-serology. (DOCX)

S4 Table General characteristics and treatment outcomes analyzed by gender (Male/Female). (DOCX)

S5 Table STROBE statement. (DOC)

## References

[1] Wassilew N, Hoffman H, Andrejak C, Lange C. Pulmonary disease caused by non- tuberculous mycobacteria. Respiration 2016;91: 386–402. doi: 10.1159/000445906 27207809 10.1159/000445906

[2] Tortoli E. Clinical manifestations of nontuberculous mycobacteria infections. Clin Microbiol Infect 2009;15:906–910. doi: 10.1111/j.1469-0691.2009.03014.x 19845702 10.1111/j.1469-0691.2009.03014.x

[3] Hoefsloot W, van Ingen J, Andrejak C, Angeby K, Bauriaud R, Bemer P, Beylis N, Boeree MJ, Cacho J, Chihota V, et al; Nontuberculous Mycobacteria Network European Trials Group. The geographic diversity of nontuberculous mycobacteria isolated from pulmonary samples: an NTM-NET collaborative study. Eur Respir J 2013;42:1604–1613. doi: 10.1183/09031936.00149212 23598956 10.1183/09031936.00149212

[4] Griffith DE, Aksamit T.R. Understanding nontuberculous mycobacterial lung disease: it’s been a long time coming. F 1000 Research 2016;5:2797 doi: 10.12688/f1000research.9272.1 27990278 10.12688/f1000research.9272.1 PMC5133682

[5] Aksamit T.R., Philey JV, Griffith DE. Nontuberculous mycobacterial (NTM) lung disease: the top ten essentials. Respiratory Medecine 2014;108: 417–425. doi: 10.1016/j.rmed.2013.09.014 24484653 10.1016/j.rmed.2013.09.014

[6] Van Ingen J, Bendien SA, De Lange WCM, Hoefsloot W, Dekhuijzen PNR, Boeree MJ, van Soolingen D. Clinical relevance of non-tuberculous mycobacteria isolated in the Nijmegen-Arnhem region, The Netherlands. Thorax 2009;64:502–506. doi: 10.1136/thx.2008.110957 19213773 10.1136/thx.2008.110957

[7] Griffith DE, Aksamit T, Brown-Eliott BA, Catanzaro A, Daley C, Gordin F, Holland SM, Horsburgh R, Huitt G, Iademarco MF. An official ATS/IDSA statement: diagnosis, treatment, and prevention of nontuberculous mycobacterial diseases. Am J Respir Crit Care Med 2007;175:367–416.[Published erratum appears in Am J Respir Crit Care Med 2007;175:744–745.] doi: 10.1164/rccm.200604-571ST 17277290 10.1164/rccm.200604-571ST

[8] Huang JH, Kao PN, Adi V, Ruoss SJ. Mycobacterium avium- intracelulare pulmonary infection in HIV-negative patients without preexisting lung disease: diagnostic and management limitations. Chest 1999; 115:1033–1040. 10208205 10.1378/chest.115.4.1033

[9] Fleshner M, Olivier KN, Shaw PA, Adjemian J, Strollo S, Claypool RJ, Folio L, Zelazny A, Holland SM, Prevost DR. Mortality among patients with pulmonary non-tuberculous mycobacteria disease. Int J Tuberc Lung Dis 2016; 20(5):582–587. doi: 10.5588/ijtld.15.0807 27084809 10.5588/ijtld.15.0807 PMC6660916

[10] Ballarino GJ, Olivier KN, Claypool RG, Holland SM, Prevost DR. Pulmonary non tuberculous mycobacterial infections: antibiotic treatment and associated cost. Respir Med 2009; 103:1448–1455. doi: 10.1016/j.rmed.2009.04.026 19467851 10.1016/j.rmed.2009.04.026 PMC2739259

[11] Andrejak C, Thomsen Vϴ, Johansen IS, RIIS A, Benfield TL, Duhaut P, Sorensen HT, Lescure FX, Thomsen R W. Non tuberculous pulmonary mycobacteriosis in Denmark: Incidence and Prognostic factors. Am J Respir Crit,Care Med 2010; 181:514–521. doi: 10.1164/rccm.200905-0778OC 20007929 10.1164/rccm.200905-0778OC

[12] Hayashi M, Takayanagi N, kanauchi T, Miyahara Y, Yanagisawa T, Sugita Y. Prognostic factors of 634 HIV-negative patients with *Mycobacterium avium complex* lung disease. Am J Respir Crit Care Med 2002; 185:575–83. doi: 10.1164/rccm.201107-1203OC 22199005 10.1164/rccm.201107-1203OC

[13] Marras TK, Chedore P, Ying AM, Jamieson F. Isolation prevalence of pulmonary non tuberculous mycobacteria in Ontario,1997–2003. Thorax 2007; 62:661–666. doi: 10.1136/thx.2006.070797 17311842 10.1136/thx.2006.070797 PMC2117272

[14] Kotilainen H, Valtonen V, Tukianen P, Poussa T, Eskola J, Järvinen A. prognostic value of American Thoracic Society criteria for non-tuberculous mycobacterial disease: retrospective analysis of 120 cases with four years follow up. Scand J Infect Dis 2013; 45:194–202. doi: 10.3109/00365548.2012.722227 23039965 10.3109/00365548.2012.722227

[15] Winthrop KL, McNelley E, Kendall B, Marshall-Olson A, Morris C, Cassidy M, Saulson A, Hedberg K. Pulmonary nontuberculous mycobacterial disease prevalence and clinical features: An emerging public health disease. Am J Respir Crit Care Med 2010; 182:977–982. doi: 10.1164/rccm.201003-0503OC 20508209 10.1164/rccm.201003-0503OC

[16] Jankovic M, Sabol I, Zmak L, Jankovic VK, Jakopovic M, Obravac M, Ticac B, Bulat LK, Popovic S, Marekovic I, Samarzija M, Van Ingen J. Microbiological criteria in non-tuberculous mycobacteria pulmonary disease: a tool for diagnosis and epidemiology. Int J Tuberc Lung Dis 2016; 20: 934–940. doi: 10.5588/ijtld.15.0633 27287647 10.5588/ijtld.15.0633

[17] Streit E, Millet J, Rastogi N. Non tuberculous mycobacteria in Guadeloupe, Martinique and French Guiana from 1994 to 2012. Tuberc Res Treat 2013; 2013:472041 doi: 10.1155/2013/472041 24455239 10.1155/2013/472041 PMC3880762

[18] Dailloux M, Abalain ML, Laurain C, Lebrun L, Loos-Ayav C, Lozniewski A, Maugein J. French Mycobacteria Study Group. Respiratory infections associated with nontuberculous mycobacteria in non-HIV patients. Eur Respir J 2006; 28:1211–1215. doi: 10.1183/09031936.00063806 17138678 10.1183/09031936.00063806

[19] Boyle DP, Zembower TR, Reddy S, Qi C. Comparison of clinical features virulence and relapse among *Mycobacterium avium complex* species. Am J Respi Crit Care Med 2015; 191:1310–1317. doi: 10.1164/rccm.201501-0067OC 25835090 10.1164/rccm.201501-0067OC

[20] Park HK, Koh W-J, Shim TS, Kwon OJ. Clinical characteristics and treatment outcomes of *Mycobacterium* kansasii lung disease in Korea. Yonsei Med J 2010; 51: 552–556. doi: 10.3349/ymj.2010.51.4.552 20499421 10.3349/ymj.2010.51.4.552 PMC2880268

[21] Prince DS, Peterson DD, Steiner RM, Gottlieb JE, Scott R, Israel HL, Fiqueroa WG, Fish JE. Infection *with Mycobacterium avium complex* in patients without predisposing conditions. N Engl J Med 1989; 321:863–868. doi: 10.1056/NEJM198909283211304 2770822 10.1056/NEJM198909283211304

[22] Kobashi Y, Matsushima T. The Microbiological and clinical effects of combined therapy according to guidelines on the treatment of pulmonary *Mycobacterium avium complex* disease in Japan—including a follow-up study. Respiration 2007; 74(4):394–400. doi: 10.1159/000095674 16954651 10.1159/000095674

[23] Ferro BE, Van Ingen J, Wattenberg M, Soolingen DV, Mouton JW. Time-Kill kinetics of slowly growing mycobacteria common in pulmonary disease. Antimicrob Chemother 2015;70(10):2838–43. doi: 10.1093/jac/dkv180 26142475 10.1093/jac/dkv180

[24] Egelund EF, Fennelly KP, Peloquin CA. Medications and monitoring in non-tuberculous mycobacteria infections. Clin Chest Med 2015; 36: 55–66. doi: 10.1016/j.ccm.2014.11.001 25676519 10.1016/j.ccm.2014.11.001

[25] Rastogi N, Goh KS, David HL. Enhancement of drug susceptibility of *Mycobacterium avium* by inhibitors of cell envelope synthesis. Antimicrob Agents Chemother 1990;34:759–764. 2360816 10.1128/aac.34.5.759 PMC171687

[26] Rastogi N, Labrousse V. Extracellular and intracellular activities of clarithromycin used alone and in association with ethambutol and rifampin against *Mycobacterium avium complex*. Antimicrob Agents Chemother 1991;35:462–470. . 1828135 10.1128/aac.35.3.462 PMC245033

[27] David HL, Rastogi N, Clavel-Sérès S, Clément F, Thorel MF. Structure of the cell envelope of *Mycobacterium avium*. Zentralbl Bakteriol Mikrobiol Hyg A. 1987;264:49–66. 3630478 10.1016/s0176-6724(87)80124-4

[28] Rastogi N, Frehel C, Ryter A, Ohayon H, Lesourd M, David HL. Multiple drug resistance in *Mycobacterium avium*: is the wall architecture responsible for exclusion of antimicrobial agents? Antimicrob Agents Chemother 1981;20:666–677. 6798925 10.1128/aac.20.5.666 PMC181770

[29] Van Ingen J, Boeree MJ, Soolingen DV, Mouton JW. Resistance mechanisms and drug susceptibility testing of nontuberculous mycobacteria. Drug Resistance uptades 2012; 15: 149–161. doi: 10.1016/j.drup.2012.04.001 22525524 10.1016/j.drup.2012.04.001

[30] Rastogi N. Recent observations concerning structure and function relationship in the mycobacterial cell envelope: elaboration in a model in terms of mycobacterial pathogenicity, virulence and drug-resistance. Res Microbiol 1991;142:464–476. 1871434 10.1016/0923-2508(91)90121-p

[31] Hoffner SE, Kratz M, Olson-liljequist B, Svenson SB, Kallenius G. In vitro synergistic activity between ethambutol and fluorinated quinolones against *Mycobacterium avium complex*. J Antimicrobiol Chemother 1989; 24: 317–324. 2808189 10.1093/jac/24.3.317

[32] Rastogi N, Labrousse V, Bryskier A. Intracellular activities of roxithromycin used alone and in association with other drugs against *Mycobacterium avium complex* in human macrophages. Antimicrob Agents Chemother 1995;39:976–978. 7786006 10.1128/aac.39.4.976 PMC162664

[33] Kasperbauer SH, Daley CL. Diagnosis and treatment of infections due to *Mycobacterium avium complex*. Semin Respir Crit Care Med 2008;29:569–576. doi: 10.1055/s-0028-1085708 18810690 10.1055/s-0028-1085708

[34] Griffith DE, Brown-Elliott BA, Shepherd S, McLarty J, Griffith L, Wallace RJ Jr. Ethambutol ocular toxicity in treatment regimens for *Mycobacterium avium complex* lung disease. Am J Respir Crit Care Med. 2005;172:250–253. doi: 10.1164/rccm.200407-863OC 15860751 10.1164/rccm.200407-863OC

[35] Lam PK, Griffith DE, Aksamit TR, Ruoss SJ, Garay SM, Daley CL, Catanzaro A. Factors related to response to intermittent treatment of *Mycobacterium avium complex* lung disease. Am J Respir Crit Care Med. 2006;173:1283–1289. doi: 10.1164/rccm.200509-1531OC 16514112 10.1164/rccm.200509-1531OC

[36] Rossi M, Flepp M, Telenti A, Shiffer V, Egloff N, Bucher H, Vernazza P, Bernasconi E, Weber R, Rickenbach M, Furrer H. Swiss HIV Cohort Study. Declining incidence, improved prognosis, and discontinuation of maintenance therapy. Swiss Med Wkly 2001;131(31–32):471–477. 11641970 10.4414/smw.2001.09728

[37] Griffith DE, Lange C. When is a non-tuberculous mycobacterial infection a pulmonary disease? Int J Tuberc lung Dis 2016; 20(7): 855–856. doi: 10.5588/ijtld.16.0243 27287633 10.5588/ijtld.16.0243

