# Supplementary material for: Predictive factors for a one-year improvement in nontuberculous mycobacterial pulmonary disease: An 11-year retrospective and multicenter study
Source: PLoS Negl Trop Dis. 2017 Aug 7;11(8):e0005841. doi: 10.1371/journal.pntd.0005841 (PMC5560745; doi:10.1371/journal.pntd.0005841)
Supplement: S1 Table — (DOCX) [file pntd.0005841.s001.docx]

**S1 table.** Treatment of non-tuberculous mycobacterial pulmonary disease (NTM-PD)

| Mycobacteria | Therapeutic regimen usually used in this study |
| --- | --- |
| M. *avium* Complex | 3 drugs Per os (P.O.) :clarithromycin , rifampicin, ethambutol |
| M. *abscessus complex* | Intensive phase : clarithromycin, ( in absence of inducible macrolide resistance) , amikacin plus imipenem , continuation phase :  3 drugs Per os (P.O.) : clarithromycin, (in absence of inducible macrolide resistance) + ciprofloxacin and linezolid |
| *M. kansasii* | 3 drugs Per os (P.O.) : rifampicin, ethambutol, isoniazid |
| *M. xenopi* | 3 drugs Per os (P.O.) : clarithromycin, rifampicin, ethambutol |
| *M. simiae* | Amikacin (intensive case only) and 2 drugs Per os (P.O.) : clarithromycin, trimethoprim/sulfamethoxazole |

Drugs for a Therapeutic regimen should be based on drugs susceptibility testing results
