# Supplementary material for: Predictive factors for a one-year improvement in nontuberculous mycobacterial pulmonary disease: An 11-year retrospective and multicenter study
Source: PLoS Negl Trop Dis. 2017 Aug 7;11(8):e0005841. doi: 10.1371/journal.pntd.0005841 (PMC5560745; doi:10.1371/journal.pntd.0005841)
Supplement: S2 Table — (DOCX) [file pntd.0005841.s002.docx]

**S2 table.** The general characteristics and treatment outcome of patients infected with *mycobacterium avium* complex (MAC).

| Mycobacterial Species = *Mycobacterium avium complex (MAC****)*** | Total  patients  n=55 (%) | Patients with Improved status  n =26 (47.2%) | Patients with unimproved status  n=29(52.7%) | P value |
| --- | --- | --- | --- | --- |
| ***Age (mean ± SD),years*** | 50.0 ±-20 | 42.8 ±19.6 | 56.4 ±18.4 | 0.04 |
| ***Sex*** |  |  |  | 0.33 |
| *Male* | 40(72.7) | 21 (80.7) | 19 (65.5) |  |
| *Female* | 15(27.2) | 5 (19.2) | 10 (34.4) |  |
| ***Respiratory history*** |  |  |  | 0.75 |
| *Yes* | 34(61.8) | 15(57.6) | 19 (65.5) |  |
| No | 21(38.1) | 11 (42.3) | 10 (34.4) |  |
| ***Respiratory disease*** |  |  |  | 0.27 |
| - Cystic fibrosis | 11(20.0) | 8 (30.7) | 3(10.3) |  |
| - Bronchiectasis | 14(25.4) | 5 (19.2) | 9 (31.0 |  |
| - Chronic obstructive pulmonary disease | 9(16.3) | 2 (7.6) | 7 (24.1) |  |
| ***HIV patients*** | 17(30.9) | 11(42.3) | 6(20.6) | 0.10 |
| ***Respiratory symptoms*** |  |  |  |  |
| Cough | 31(56.3) | 16(61.5) | 15(51.7) | 0.35 |
| *Sputum*  ***Radiology results*** | 31(56.3) | 14(53.8) | 17(58.6) | 0.81 |
| Bronchiectasis | 31(56.3) | 14 (53.8) | 17 (44.8) | 0.80 |
| Nodular opacities | 14(25.4) | 5 (19.2) | 9(31.0) | 0.55 |
| cavities | 9(16.3) | 3(11.0) | 6(20.6) | 0.49 |
| **ATS/ IDSA criteria** | 34(61.8) | 17(65.3) | 17(58.6) | 0.83 |
| ***Treatment*** | 34 (61.8) | 18(69.2) | 16 (55.1) | 0.66 |
| ***Duration of treatment (month)*** | 8.4 ±5.9 | 7.5 ±6.9 | 6.2±3.6 | 0.06 |
| **Clarithromycin** (months) |  | 9.6 ±6.8 | 4.6±3..8 | 0.02 |
| **Rifampin** (months) |  | 5.9 ±6.4 | 4.6±3.2 | 0.25 |
| **Ethambutol** (months) |  | 5.7 ±6.4 | 4.5±3.7 | 0.10 |
|  |  |  |  |  |
| **Negative cultures at one year** | 33 (60.0) | 26(100) | 7(21) | 0.001 |
| **Outcome of patients** |  |  |  |  |
| Deaths at one year | 9 (16.3) | 0 (0.0) | 9 (31.0) | 0.001 |
|  |  |  |  |  |

**Improved status at 1-year**: Patients improved clinically and radiological and their microbiological samples were negative. **Unimproved status at 1-year:** The patients did not improve their clinical state or their radiological lesions or their microbiological samples did not negative.

**ATS/IDSA:** American Thoracic Society and the Infectious Disease Society of America

**SD** : standard deviation
