# Supplementary material for: Predictive factors for a one-year improvement in nontuberculous mycobacterial pulmonary disease: An 11-year retrospective and multicenter study
Source: PLoS Negl Trop Dis. 2017 Aug 7;11(8):e0005841. doi: 10.1371/journal.pntd.0005841 (PMC5560745; doi:10.1371/journal.pntd.0005841)
Supplement: S3 Table — (DOCX) [file pntd.0005841.s003.docx]

**S3 table .** The general characteristics and treatment outcome of patients with positive HIV-serology.

| **Patients with positive**  **HIV-serology** | Total  patients  n=29 (%) | Patients with improved status  n =17 (58.6%) | Patients with unimproved status n=12 (41.3%) | P value |
| --- | --- | --- | --- | --- |
| ***Age (mean ± SD),years*** | 49.2 ±-19.4 | 46.1±19.2 | 53.1±19.1 | 0.05 |
| ***Sex*** |  |  |  | 0.59 |
| *Male* | 22(75.8) | 14 (82..3) | 8 (66.6) |  |
| *Female* | 7(24.1) | 3 (17.6) | 4( 33.3) |  |
| ***Respiratory history*** |  |  |  | 0.34 |
| *yes* | 6(20.6) | 2(11.7) | 4 (33.3) |  |
| No | 23(79.3) | 15(88.2) | 8 (66.6) |  |
| ***Respiratory symptoms*** |  |  |  |  |
| Cough | 18(62.0) | 11(64.7) | 7 (58.3) | 0.90 |
| *Sputum*  ***Radiology results*** | 9(31.0) | 7(41.1) | 2(16.6) | 0.43 |
| Bronchiectasis | 5(17.2) | 2 (11.7) | 3 (25.0) | 0.63 |
| Nodular opacities | 9(31.0) | 6 (35.2) | 3(25.0) | 0.25 |
| Cavities | 7(24.1) | 1(5.8) | 6(50.0) | 0.08 |
| **Mycobacterial species** |  |  |  |  |
| *M. avium* complex (MAC) | 17(58.6) | 11(64.7) | 6 (50.0) | 0.76 |
| *M. simiae* | 4 (13.7) | 2 (11.7) | 2 (16.6) | 0.99 |
| *M. fortuitum* | 3 (10.3) | 2(11.7) | 1 (8.3) | 0.98 |
| *M. kansasii* | 2(6.8) | 0(0.0) | 2 (16.6) | 0.19 |
| **ATS/ IDSA criteria** | 13(44.8) | 8(47.0) | 5(41.6) | 0.99 |
| **ATS/IDSA microbiologic criteria** | 15 (51.7) | 9 (52.9) | 6 (50.0) | 0.96 |
| **CD4<200** | 23 (79.3) | 14(82.3) | 9 (75.0) | 0.98 |
| ***Treatment (n, %)*** | 16(55.1) | 13(76.4) | 3 (25.0) | 0.04 |
|  |  |  |  |  |
| ***Negative cultures at one year*** | 17(58.2) | 17(100) | 0(0.0) | 0.001 |
| ***Outcome*** |  |  |  |  |
| Deaths at one year | 5 (17.2) | 0 (0.0) | 5(41.6) | 0.04 |
|  |  |  |  |  |

**Improved status at 1-year**: Patients improved clinically and radiological and their microbiological samples were negative. **Unimproved status at 1-year:** The patients did not improve their clinical state or their radiological lesions or their microbiological samples did not negative.

**ATS/IDSA:** American Thoracic Society and the Infectious Disease Society of America

**SD** :standard deviation
