# Supplementary material for: Predictive factors for a one-year improvement in nontuberculous mycobacterial pulmonary disease: An 11-year retrospective and multicenter study
Source: PLoS Negl Trop Dis. 2017 Aug 7;11(8):e0005841. doi: 10.1371/journal.pntd.0005841 (PMC5560745; doi:10.1371/journal.pntd.0005841)
Supplement: S4 Table — (DOCX) [file pntd.0005841.s004.docx]

**S4 table.** General characteristics and treatment outcomes analyzed by gender (Male/Female).

|  | Total  patients  n=119 (%) | Male  Patients  N=80 (67.2 %) | Female  Patients  N=39 (32.8%) | P value |
| --- | --- | --- | --- | --- |
| ***Age (mean ± SD ),years*** | 50.4 ±-19.4 | 48.1 ±19.2 | 55.0±19.0 | 0.04 |
| ***Respiratory history*** |  |  |  |  |
| *yes* | 70(58.8.) | 44(55.0) | 26 (66.6) | 0.63 |
| ***Respiratory disease***  *Cystic fibrosis*  *Bronchiectasis*  *Chronic obstructive pulmonary disease* | 23(19.3)  23(19.3)  18 (15.1) | 16 (20)  6 (7.5)  16 (20) | 7 (17.9)  17 (43.5)  2 (5.1) | 0.004 |
| ***Respiratory symptoms*** |  |  |  |  |
| Cough | 79(66.4) | 50(62.5) | 29(74.3) | 0.64 |
| *Sputum*  ***Radiology results*** | 61(51.3) | 30(37.5) | 31(79.4) | 0.02 |
| Bronchiectasis | 54(45.4) | 29 (36.2) | 25 (64.1) | 0.04 |
| Nodular opacities | 24(20.1) | 15 (18.7) | 9(23.0) | 0.45 |
| Cavities | 26(21.8) | 20(25.0) | 6(15.3) | 0.05 |
| **Mycobacterial species** |  |  |  |  |
| *M. avium* complex (MAC) | 55(46.2) | 40 (50.0) | 15 (38.4) | 0.48 |
| *M. abscessus complex* | 17 (14.3) | 10 (12.5) | 7 (17.9) | 0.58 |
| *M. fortuitum* | 16 (13.4) | 10(12.5) | 6(15.3) | 0.77 |
| *M. simiae* | 11(9.2) | 6(7.5) | 5(12.8) | 0.44 |
| **ATS/ IDSA criteria** | 68(57.1) | 42(52.5) | 26(66.6) | 0.52 |
| **ATS/IDS microbiologic criteria** | 76(63.9) | 50 (62.5) | 26 (66.6) | 0.87 |
| ***Treatment (n, %)*** | 63(52.9) | 45(56.2) | 18 (46.1) | 0.61 |
| **Negative cultures at one year** | 61 (51.2) | 44(55) | 17(43.5) | 0.61 |
| ***Status*** |  |  |  | 0.04 |
| ***improved*** | 46 (38.6) | 36(45.0) | 10 (25.6) |  |
| **unimproved**  ***Patient Outcome*** | 73 (61.3) | 44 (55.0) | 29 (74.3) |  |
| Deaths at one year | 17 (14.2) | 13 (16.2) | 4(10.2) | 0.58 |
|  |  |  |  |  |

**Improved status at 1-year**: Patients improved clinically and radiological and their microbiological samples were negative. **Unimproved status** **at 1-year:** The patients did not improve their clinical state or their radiological lesions or their microbiological samples did not negative.

**ATS/IDSA:** American Thoracic Society and the Infectious Disease Society of America

**SD**: standard deviation
